# Supplementary figures and images for: Disruption of de novo purine biosynthesis in Pseudomonas fluorescens Pf0-1 leads to reduced biofilm formation and a reduction in cell size of surface-attached but not planktonic cells
Source: PeerJ. 2016 Jan 4;4:e1543. doi: 10.7717/peerj.1543 (PMC4715448; doi:10.7717/peerj.1543)

Supporting Figure S1

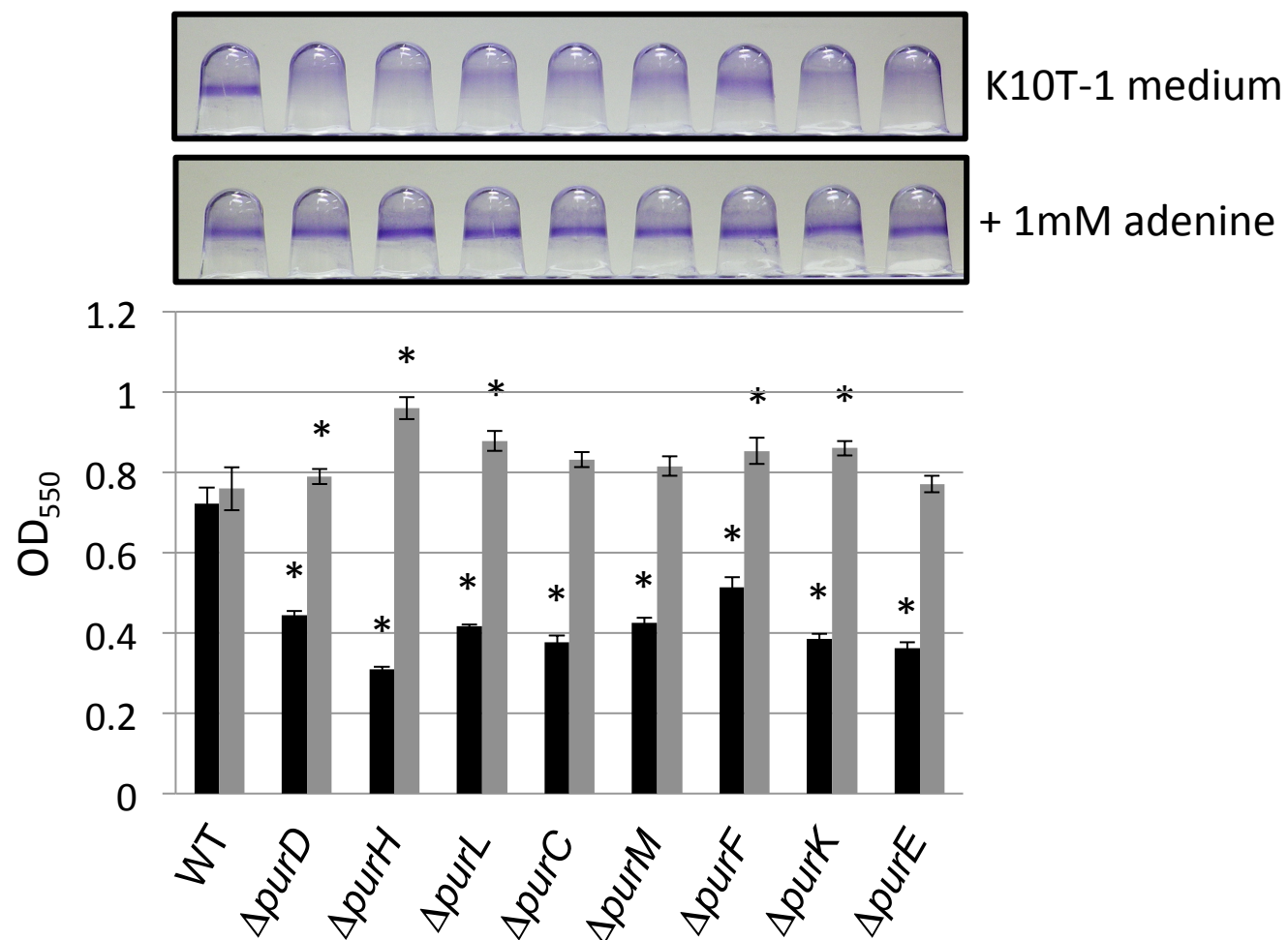

Supplement: Supplemental Information 1 — The black and light gray bars show biofilm formations in the absence and presence of 1 mM adenine, respectively. Data are the mean absorbance at 550nm ± SD (n = 7). The asterisks (∗) indicate statistically significant differences in absorbance at 550 nm (OD550) relative to that of WT (P < 0.01 in two-tailed Student’s t-test assuming equal variance). The representative images for the biofilms are shown above the figure. The experiments were done duplicate. [file peerj-04-1543-s001.pdf]
